# Hyperactivation of the PI3K pathway in inborn errors of immunity: current understanding and therapeutic perspectives

**DOI:** 10.1093/immadv/ltae009

**Published:** 2024-11-07

**Authors:** Hanna IJspeert, Virgil A S H Dalm, Menno C van Zelm, Emily S J Edwards

**Affiliations:** Laboratory Medical Immunology, Department of Immunology, Erasmus MC, University Medical Center, Rotterdam, The Netherlands; Academic Center for Rare Immunological Diseases (RIDC), Erasmus MC, University Medical Center, Rotterdam, The Netherlands; Laboratory Medical Immunology, Department of Immunology, Erasmus MC, University Medical Center, Rotterdam, The Netherlands; Academic Center for Rare Immunological Diseases (RIDC), Erasmus MC, University Medical Center, Rotterdam, The Netherlands; Division of Allergy & Clinical Immunology, Department of Internal Medicine, Erasmus MC, University Medical Center, Rotterdam, The Netherlands; Laboratory Medical Immunology, Department of Immunology, Erasmus MC, University Medical Center, Rotterdam, The Netherlands; Allergy and Clinical Immunology Laboratory, Department of Immunology, School of Translational Medicine, Monash University, Melbourne, VIC, Australia; The Jeffrey Modell Diagnostic and Research Centre for Primary Immunodeficiencies, Melbourne, VIC, Australia; Department of Allergy, Immunology and Respiratory Medicine, Central Clinical School, Alfred Hospital, Melbourne, VIC, Australia; Allergy and Clinical Immunology Laboratory, Department of Immunology, School of Translational Medicine, Monash University, Melbourne, VIC, Australia; The Jeffrey Modell Diagnostic and Research Centre for Primary Immunodeficiencies, Melbourne, VIC, Australia; Department of Allergy, Immunology and Respiratory Medicine, Central Clinical School, Alfred Hospital, Melbourne, VIC, Australia

**Keywords:** PI3K pathway, activated PI3K delta syndrome, APDS-like, immunodeficiency, immunotherapy

## Abstract

The phosphoinositide-3-kinase (PI3K) pathway function is crucial to the normal development, differentiation, and function of immune cells including B, T, and NK cells. Following the description of two cohorts of patients with an inboirn error of immunity (also known as primary immunodeficiency) with gain-of-function variants in the *PIK3CD* gene a decade ago, the disease entity activated PI3K delta syndrome (APDS) was named. Since then, many more patients with *PIK3CD* variants have been described, and loss-of-function variants in *PIK3R1* and *PTEN* have also been linked to APDS. Importantly, the availability of small molecules that inhibit the PI3K pathway has enabled targeted treatment of APDS patients. In this review, we define (i) the PI3K pathway and its role in inborn errors of immunity; (ii) the clinical and immunological presentation of APDS1 (*PIK3CD* GOF), APDS2 (*PIK3R1* LOF), and related disorders; (iii) Diagnostic approaches to identify and functionally validate the genetic causes of disease; (iv) therapeutic interventions to target PI3K hyperactivation; and finally (v) current challenges and future perspectives that require attention for the optimal treatment of patients with APDS and APDS-L diseases.

## Graphical Abstract

**Graphical Abstract:**
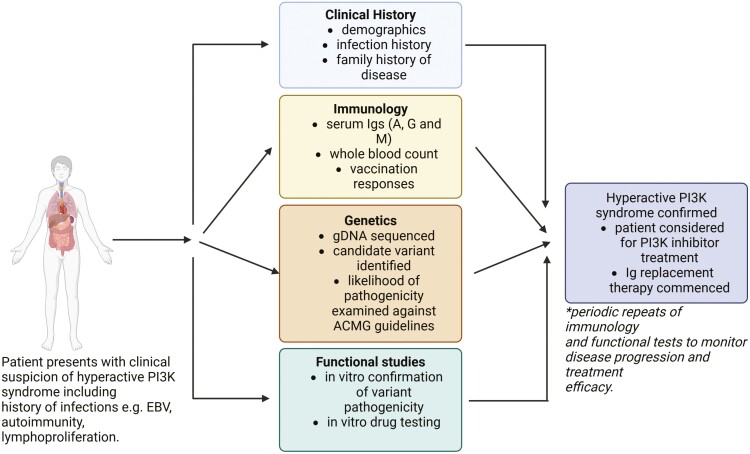

## Introduction

Inborn errors of immunity (IEI) is a rare group of inherited disorders that are characterized by increased susceptibility to severe and recurrent infections and non-infectious complications including autoimmunity, haematological malignancies, auto-inflammatory complications, and gastrointestinal disease [1]. Predominantly antibody deficiency (PAD) is the most common IEI with a global prevalence of 1/25 000 [2–4]. Still in less than 30% of PAD patients, a causative, germline gene variant has been identified. In these analyses, defects have been identified in genes encoding components of the B-cell receptor (BCR) signalling cascade including genes involved in the phosphoinositide 3-kinase (PI3K) pathway, e.g. *BTK*, *SYK*, *PTEN*, *PIK3R1*, *PIK3CD*, and *PIK3CG* [1, 5].

## The PI3K pathway signal cascade and its function

The PI3K pathway is evolutionarily conserved and is important for numerous cellular functions. In particular, the PI3K pathway has been shown to regulate immune cell development, differentiation, activation, survival, metabolism, and function. PI3Ks are lipid kinases that play a pivotal role in cellular biology [6–8]. While three classes of PI3K exist, the predominant type involved in lymphocyte signalling is Class IA PI3K, which are heterodimers comprising of a catalytic (p110α, β, or δ) and a regulatory subunit (p85α, β, or p50α, p55α) [6–9]. Of note, p110δ is predominantly expressed in leukocytes [6, 8, 10], whereas p110α and β are ubiquitously expressed [6, 8].

**Figure 1.**
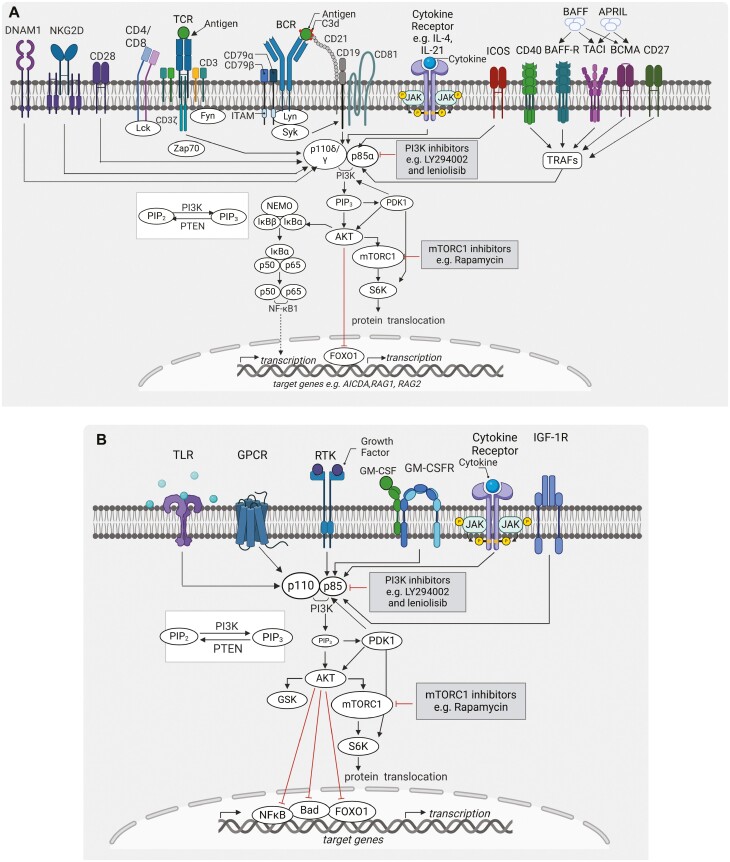
Schematic of the PI3K pathway in immune (B cells, T cells) and non-immune cells (neurons of the brain). Class I PI3K signalling pathways in A. B and T cells and C. neurons. The schematics show a simplified class IA PI3K signalling pathways in immune and non-immune cells. In B cells and T cells, are activated downstream of a multitude of cell surface receptors (e.g. BCR, TCR, cytokine receptors, CD27, CD28, and ICOS). In neurons, PI3K is activated upon ligation of cell surface receptors including RTKs, cytokines and GPCRs. Once activated, PI3K phosphorylates PIP2 (PI(4,5)P2) to PIP3 (PI(3,4,5)P3) inducing downstream activation of AKT, a central hub of signalling in these cells. This results in phosphorylation of downstream effector proteins including mTORC1, ribosomal S6 and GSK, as well as transcription factor FOXO leading to nuclear exclusion. Phosphorylation of mTORC1 and S6 regulates cell biology including proliferation, survival, growth and metabolism. While inhibition of FOXO reduces transcription of genes including AICDA, RAG1, and RAG2. Created with BioRender.com ©.

Class IA PI3K is integral in the propagation of signalling downstream of cell surface receptors, including the BCR, the T-cell receptor (TCR), Toll-like receptors (TLRs), G-coupled receptors (GPCRs), cytokine receptors, and BCR or TCR co-receptors (**Fig. 1**). In addition, PI3K activation can be amplified by co-engagement of receptors, most prominently CD19, but also BAFF-R and CD40 on B cells [7–9, 11]. Furthermore, upon ligand binding, CD28 and ICOS induce PI3K signalling in T cells (**Fig. 1A**) [6–8, 11–14].

In absence of receptor stimulation, the regulatory subunit (p85α) inhibits catalytic activity of p110δ. The p85α N-terminal Src-homology 2 (nSH2) and inter-SH2 (iSH2) domains interact with the core, helical and catalytic domains of p110δ. Separately, the iSH2 domain of p85α binds the N-terminal p110δ adaptor binding domain. Together, these crucial interactions result in inhibition of the catalytic activity of p110δ [15].

Upon engagement of a cell surface receptor, tyrosine kinases including Fyn and Lck, phosphorylate critical tyrosine residues within YxxM motifs, which reside in the cytoplasmic domain of cell surface receptors or in adaptor proteins. This phosphorylation causes receptor dimerization and recruitment of p85α (or p50α or p55α) to phosphorylated YxxM motif via its SH2 domains (**Fig. 1A**) [6–8]. Recruitment of p85α induces a conformational change in the p85α- p110δ heterodimer relieving inhibition on p110δ causing recruitment of PI3K to the plasma membrane. Activated PI3K then phosphorylates phosphatidylinositol bisphosphate (PIP_2_) to phosphatidylinositol (3,4,5)P_3_ (PIP_3_). PIP_3_ binds and recruits AKT (also known as protein kinase B) to the plasma membrane [9, 15, 16], where it is phosphorylated at conserved serine residues by mTORC2, which exposes conserved threonine residues for phosphorylation by PDK1. For optimal AKT activity, phosphorylation of both threonine and serine residues is required. Phosphorylated AKT then dissociates from PIP_3_ going on to phosphorylate a large number of effector molecules including mTORC1 (mTOR) and ribosomal protein S6, and inducing FOXO (forkhead box O) transcription factor downregulation (**Fig. 1A**) [16].

Regulation of PI3K-mediated activation is opposed by lipid phosphatases, phosphatase and tensin homolog (PTEN), and SH2-containing inositol 5ʹ-phosphatase (SHIP) (**Fig. 1A**). PTEN and SHIP dampen signalling by dephosphorylating PIP_3_ leading to the production of PIP_2_, which causes inhibition of AKT. This balancing of PI3K signals modulates signalling critical for immune cell development, survival, differentiation, metabolism, and effector function [6, 8, 11, 17].

Class IA PI3K is also expressed in neurons of the brain and operates downstream of receptors including receptor tyrosine kinases (RTKs), GPRs, granulocyte-macrophage colony stimulating factor receptor (GM-CSFR), and insulin-like growth factor 1 receptor (IGF-1R) (**Fig. 1B**). Here, the mechanism as described is propagated PI3K signalling upon receptor ligation to regulate signal transduction, which is important for neuronal processes including neuronal cell proliferation, apoptosis, and metabolism (**Fig. 1B**) [18].

## Disorders causing dysregulated PI3K signalling

### Hyperactive PI3K pathway function

Activated PI3Kδ syndrome (APDS) is caused by variants in the *PIK3CD* or *PIK3R1* genes (**Table 1**), resulting in hyperactivity of the AKT-mTOR-PI3K pathway, as well as progressive immunodeficiency and/or dysregulation.

**Table 1.** Variants in genes causing defects in PI3K Pathway activity

*Gene Symbol*	OMIM gene	Disease	OMIM phenotype	Inheritance	HGNC ID	Gene Name (HGNC)	MANE select RefSeq transcript	MANE select RefSeq protein	Variants identified to date	References
*PIK3CD*	602839	APDS1/PASLI/immunodeficiency 14A with lymphoproliferation	615513	AD	8977	Phosphoinositide-3-kinase catalytic subunit delta	NM_005036.5	NP_005017.3	E81K, G124D, S312C, N334K, R405C, C416R, E525A, E525AK, R929C, E1021K,	[19–29]
*PIK3CD*	602839	p110δ deficiency/ immunodeficiency 14B	619281	AR	8977	Phosphoinositide-3-kinase catalytic subunit delta	NM_005036.5	NP_005017.3	D853Gfs*20 V552Sfs*26 N853Gfs	[30–32]
*PIK3CG*	601232	PI3γ deficiency	619802	AR	8978	Phosphoinositide-3-kinase 3-kinase catalytic subunit gamma	NM_001282426.2	NP_001269355.1	R982fs+R1021P R49S+N1085S	[33, 34]
*PIK3R1*	171833	APDS2/PASL-R1/immunodeficiency 36 with lymphoproliferation	616005	AD	8979	Phosphoinositide-3-kinase regulatory subunit 1	NM_181523.3	NP_852664.1	Δ434-475 N564K	[25, 26, 28, 29, 35–44]
*PIK3R1*	171833	p85α deficiency/ Agammaglobulinemia 7	615214	AR	8979	Phosphoinositide-3-kinase regulatory subunit 1	NM_181523.3	NP_852664.1	?298X R301X	[45, 46]
*PTEN*	601728	PTEN deficiency	158350	AD	9588	Phosphatase and tensin homolog	NM_00314.8	NP_000305.3	I5fs R15fsX9 Y29X Y68C R233X	[47–49]
*SYK*	600085	SYK GOF or Immunodeficiency 82 with systemic inflammation	619381	AD	11491	Spleen-associated tyrosine kinase	NM_003177.7	NP_003168.2	P324T A353T M450I S550Y S550F	[50]
*LCP2*	601603	SLP76 deficiency or Immunodeficiency 81	619374	AR	6529	Lymphocyte cytosolic protein 2	NM_005565.5	NP_005556.1	K309fsX17 Q331Sfs+6 P190R+R204W	[51–53]

LCP2, SYK, PTEN, AR, AS.

APDS1 was first described in 2006 [54], with further variants published in 2013 [19, 20]. To date, 10 heterozygous genetic variants in *PIK3CD* (the gene encoding p110δ) have been shown to lead to a gain-of-function (GOF) effect and cause APDS1 with 85% patients harbouring the p.E1021K mutation [19–29]. (**Table 1**). A second related disorder, APDS2, due to heterozygous loss-of-function variants in *PIK3R1* (the gene encoding p85α, p55α, and p50α) was identified in 2014 [6]. Disease-causing heterozygous variants in *PIK3R1* were all found to affect a donor or acceptor splice site causing skipping of exon 11 and resulting in an in-frame deletion of amino acids 434–475, thereby representing a mutational hotspot [25, 26, 28, 29, 35–44]. Exon 11 encodes a region that is required for direct binding of p85α to p110δ. In both APDS1 and APDS2, direct binding interactions between p85α and p110δ are disrupted, reducing either p85α inhibition of p110δ activity or p85α binding to the phosphatase domain of PTEN, culminating in overactive PI3K pathway signalling [6, 8, 19, 20, 35, 37].

Furthermore, LOF variants in PTEN have been shown to result in hyperactive PI3K signalling (**Table 1**), resulting from inhibition of PTEN activity which increases PIP3:PIP2 ratios. This drive increased phosphorylated-AKT (p-AKT) and phosphorylated-ribosomal-S6 levels (p-S6) characteristic signs of APDS, thereby designating it as an APDS-like (APDS-L) disorder [47, 55]. To date, PTEN deficiency is the only disorder to be named as APDS-L. However, recently, SYK GOF has been shown to increase SYK autophosphorylation and downstream MAPK [50] and PI3K activity [56] therefore suggesting that this disorder may represent another APDS-L condition. As such, we expect that due to the number of proteins that activate PI3K signalling, over time more widespread assessment of PI3K signalling activity is likely to identify more APDS-L disorders.

Finally, it has been shown that somatic mutations in the PI3K genes including *PIK3CA* and *PIK3R1* can cause malignancies including breast, head, and neck cancer by enhancing PI3K activity. Therefore, somatic variants should also be considered as rare drivers of IEI particularly in cases where malignancies are detected [57].

### Underactive PI3K pathway function

While disorders resulting in overactivation of the PI3K pathway are the most researched, disorders resulting from homozygous variants in *PIK3CD* [30–32], *PIK3CG* (encoding p110γ) [33, 34], *PIK3R1* [45, 46], and *LCP2* (encoding Src2 homology domain 2-containing leukocyte protein of 76kDa or SLP76) [51] have been shown to result in reduced activity of this critical immune pathway, again leading to immunodeficiency and immune dysregulation. As this review focuses on overactive PI3K activity, we will not delve into the intricacies of these diseases. Unlike for APDS1 and 2 where more than 50 patients per defect have been described [19–29,35–44], fewer patients have been shown to suffer from autosomal recessive p110δ deficiency (2 kindreds) [30–32], p85α deficiency (3 patients) [45, 46], or PIK3CG deficiency (2 patients) [33, 34]. However, in all patients, loss of expression/or activity of the encoded protein in patient T cells, dendritic cells, and/or neutrophils is evident [30]. In several cases of *PIK3CD* and *PIK3CG* deficiency, p-AKT levels were shown to be reduced, indicative of impaired PI3K pathway activity [32–34].

Overall, genetic variants in components of the PI3K pathway can either enhance or impair its signalling capacity, highlighting the need for functional evaluation upon identification of a new variant in a patient with immunodeficiency.

## Clinical manifestations of PI3K-related PIDs

### APDS1 and APDS2

APDS has a heterogeneous clinical presentation (**Table 2**) that usually starts with an early-childhood onset of infections (median age 1 year) and lymphoproliferation (median age 3 years) and progresses to autoimmunity in later childhood. Malignancy can occur at any age but typically not before late childhood/early adulthood (median age 18 years) [59]. Patients may initially be diagnosed with an antibody deficiency or an autoimmune lymphoproliferative syndrome. A large study of 277 APDS patients (including both APDS1 and APDS2) reported that 96% of the patients had respiratory tract infections, with otitis media, rhinosinusitis, and ocular infections occurring in 73% of the patients [58]. Cutaneous abscesses and lymphadenitis were also reported in about 22% of the patients. Bacterial pneumonia was reported in 82% of the patients [54]. The encapsulated bacterial pathogens *Streptococcus pneumonia* and *Haemophilus influenza* were most frequently identified, although infections with *Staphylococcus aureus*, *Pseudomonas aeruginosa*, *Moraxella catarrhalis*, and *Klebsiella* species are also frequently reported [58, 64]. Sepsis is less common and reported in 10% of the APDS patients [58]. Viral infections are also reported in ~50% of patients, especially with the herpes viruses Epstein Barr Virus (EBV), cytomegalovirus (CMV), herpes simplex virus (HSV), and varicella zoster virus (VZV) [58]. Infections with fungal and parasitic pathogens are rare. Most common is oral mucocutaneous candidiasis (13% of patients), whereas diarrhoea caused by *Cryptosporidium parvum*, giardia, or toxoplasmosis has been incidentally reported [58].

**Table 2.** Clinical manifestations PI3K hyperactivation syndromes

Clinical features	APDS1	APDS2	APDS-L	SYK GOF
Recurrent respiratory infections	95% (173) [58]	99% (66) [58]	8 patients [47–49]	100% (6) [50]
Bronchiectasis	60% [26]	26% [26]	1 patient [47]	Not reported
Herpes viremia/infection	53% (102) [58]	38% (21) [58]	Not reported	Not reported
Lymphadenopathy	55.9% (100) [59]	76.6 % (49) [59]	24% (19) [60]	Not reported
Splenomegaly	50.3% (90) [59]	39.1 (25) [59]	1 patient [49]	Not reported
Hepatomegaly	34.6% (62) [59]	12.5% (8) [59]	1 patient [49]	Not reported
Malignancy	10.6 (19) [59]	18.8% (12) [59]	40% (49) [61]	33% (2) [50]
Autoimmunity	31.3% (56) [59]	20.3% (13) [59]	26.6% (21) [60]	100% (6) [50]
Enteropathy	28.5% (51) [59]	21.9% (14) [59]	47.1 % (16) (gastrointestinal lymphoid hyperplasia) [62]	100% (6) [50]
Failure to thrive/short stature	9.5% (17) [59]	45% (14) [36] 51.6% (33) [59]	2 patients [63]	33% (2) [50]

The number in brackets is the number of reported patients with a particular complication if reported.

Similar to other PAD with recurrent respiratory infections, patients with APDS are at risk for developing bronchiectasis. Besides the frequent respiratory infections that may lead to chronic inflammatory airway damage, focal nodular lymphoid hyperplasia may obstruct airways, and lead to post-obstructive bronchiectasis in APDS patients [65]. Previous reports, including from the ESID APDS registry, suggested that there might be a higher incidence of bronchiectasis in APDS1 (60%) than in APDS2 patients (26%) [24, 26, 36].

Lymphoproliferation is another hallmark of APDS, and reported in the majority of the patients (up to 86%) [26, 59]. Lymphadenopathy is most frequently reported (61%), followed by splenomegaly (47%) and hepatomegaly (29%) [59]. While lymphadenopathy seems to be more frequent in APDS2 (55.9% in APDS1 versus 76.6% in APDS2), splenomegaly (50.3% in APDS1 versus 39.1% in APDS2), and hepatomegaly (34.6% in APDS1 versus 12.5% in APDS2) are more often reported in APDS1 [26, 59]. Besides benign lymphoproliferation, APDS patients have an increased risk of malignant lymphoproliferation. Lymphomas are reported in around 14% of APDS patients, and ~25% of these patients developed multiple lymphomas [26, 59]. Of note, 17 of the 22 lymphoma cases were preceded by chronic benign lymphoproliferation, half which were EBV-associated [26, 59]. The incidence for lymphoid malignancies was higher in APDS2 versus APDS1 and the APDS2 patients in the ESID cohort also showed a high incidence of non-lymphoid malignancies [26].

In about 30% of the APDS patients, autoimmunity is reported [24, 59]. The most common autoimmune manifestations are cytopenia’s: autoimmune haemolytic anaemia (AIHA) and immune thrombocytopenia purpura (ITP). However, organ-specific autoimmunity has also been reported: autoimmune thyroiditis, glomerulonephritis, nephrotic syndrome, insulin-dependent diabetes, exocrine pancreatic insufficiency, autoimmune hepatitis, arthritis, Sjogren’s syndrome, and pericarditis [24, 26, 59]. About 25% of the APDS patients present with enteropathy (diarrhoea, malabsorption, intestinal nodular lymphoid hyperplasia) which is related to infections, and/or autoimmunity [59].

Non-immunological symptoms, such as growth impairment and syndromic characteristics, are significantly more frequent in patients with APDS2 than in APDS1 patients [26]. This is likely due to the broader expression pattern of p85α compared to p110δ protein, whereby while p110δ is predominantly expressed in lymphocytes, p85α is also expressed in cells of the lung and brain for example. Heterozygous loss-of-function mutations in *PIK3R1* can also result in SHORT syndrome, which is characterized by **S**hort stature, **H**yperextensibility of joints and/or hernias, **O**cular depression, **R**ieger anomaly and delays of **T**ooth eruptions. In contrast to APDS2, the *PIK3R1* variants associated with SHORT syndrome are typically located in the inter-SH2 or C-terminal SH2 domains of the p85α [66–69]. It is hypothesized that variants associated with SHORT syndrome lead to increased basal PI3K signalling, similar to APDS2, but that receptor stimulation of certain ligands, such as insulin, does not result in enhanced signalling [43, 67, 69]. Although heterozygous loss-of-function *PIK3R1* variants usually result in either APDS2 or SHORT syndrome, a few patients with exon 11 skipping PIKR1 variants, have been described with overlapping features [38, 43, 70].

Neurological and learning disorders have been frequently reported in APDS2 (26.6%) and APDS1 (9.5%) [59]. A recent study showed that APDS1 patients have visuomotor defects, exacerbated by autism spectrum disorder comorbidity and a mouse model for APDS1 (p110δ E1021K mice) exhibits impairments of motor behaviour, learning, and repetitive behaviour patterning [71].

A systematic review of mortality and survival rates showed that APDS patients have a shortened average lifespan compared to healthy individuals [72]. Kaplan–Meier survival analysis of 256 APDS patients showed a 30-year survival of 74%, which is similar to the 30-year survival rate of 70% of males and 68% of female in genetically undefined CVID [72, 73]. The most common causes of death for APDS1 were lymphoma and complications from haematopoietic stem cell transplantation (HSCT). All reported deaths in the APDS2 cohort (*n* = 5) were related to lymphomas [72].

### APDS-like (APDS-L) immunodeficiency

Heterozygous germline pathogenic variants in *PTEN* are associated with a range of disorders that are known under the umbrella term ‘PTEN hamartoma tumour syndrome’ (PHTS) [74] and include Cowden syndrome, Bannayan-Riley-Ruvalcaba syndrome, PTEN-related Proteus syndrome, and PTEN-related Proteus-like syndrome. PHTS is characterized by the development of multiple hamartomas with a lifetime risk for developing breast cancer of 85%, thyroid cancer of 35%, renal cell cancer of 34%, and endometrial cancer of 28% [75]. Unlike APDS1 and APDS2, lymphomas are less commonly reported in PHTS. Most of the patients with PHTS have macrocephaly (94%) [76].

PHTS was initially not linked to immunodeficiency, even though the first case report of a patient with Cowden’s disease and immunodeficiency was already published in 1981 [77]. However, several case series had reported lymphoid hyperplasia and autoimmunity in patients with PHTS. A study of 79 PHTS patients, without reported clinical immunodeficiency, showed presence of lymphoid hyperplasia in 24% and autoimmunity in 27% of the PHTS patients [60]. They presented mostly with thyroiditis and colitis. Only in 2014, after GOF variants in *PIK3CD* were described, several publications linked PHTS to recurrent infections, and the term APDS-L disease was introduced [47–49]. Still, only a few case series with in total nine patients have been reported that PHTS patients can present with recurrent infections, suggesting that the incidence of immunodeficiency in PHTS patients is low [47–49, 77]. These patients suffered mostly from recurrent respiratory tract infections and ENT infections. One patient was hospitalized for *Pneumocystis jiroveci* pneumonia at 4 months of age, one patient had candida esophagitis, and another had pulmonary aspergillus [47–49]. In contrast to patients with APDS1 or APDS2, infections with herpes viruses were not reported in APDS-L syndrome. Lymphoproliferation (hyperplasia, hepatosplenomegaly, and/or systemic poly-lymphadenopathy) has been described in a few patients that presented with PHTS and immunodeficiency. So, the clinical picture of patients with LOF variants in *PTEN* seems to be more complex than APDS1 and APDS and more frequently associated with immune dysregulation rather than recurrent infections. This might be explained by the ubiquitous expression of PTEN and its major role in cell cycle arrest, apoptosis, cell adhesion, cell migration, and cell differentiation [78].

### SYK gain-of-function

In 2021, six patients with heterozygous *SYK* gain-of-function (GOF) variants were described [50]. These patients presented with recurrent infections and very early-onset multi-organ inflammation. The infectious problems included, oral ulcers (*n* = 1), bronchitis (*n* = 1), pneumonia (*n* = 2), otitis media (*n* = 1), abscesses (*n* = 2), salmonellosis (*n* = 1), helicobacter pylori (*n* = 1) and herpes zoster infections (*n* = 1), osteomyelitis caused by Streptococcus spp. (*n* = 1), and Campylobacter gastroenteritis (*n* = 1). All patients presented with intestinal inflammation with a very early onset in half of the patients. Other organ inflammation included skin inflammation (5/6 patients), joint inflammation (4/6 patients), lung inflammation (2/6 patients), central nervous inflammation (2/6 patients), and liver inflammation (1/6 patients). Similar to APDS and APDS-L patients, the SYK GOF patients presented with lymphoproliferative disease which included hyperplasia (*n* = 2), lymphadenopathy (*n* = 3), and splenomegaly (*n* = 2). Two of the patients developed diffuse large B cell lymphoma at adulthood [50]. The authors did not report any measurements on the PI3K signalling pathway, so it is unknown if these patients have enhanced PI3K signalling like the unpublished observations by Edwards *et al*.

## Diagnostic approaches: genetic and functional testing for PI3K-pathway dysregulation

**Figure 2.**
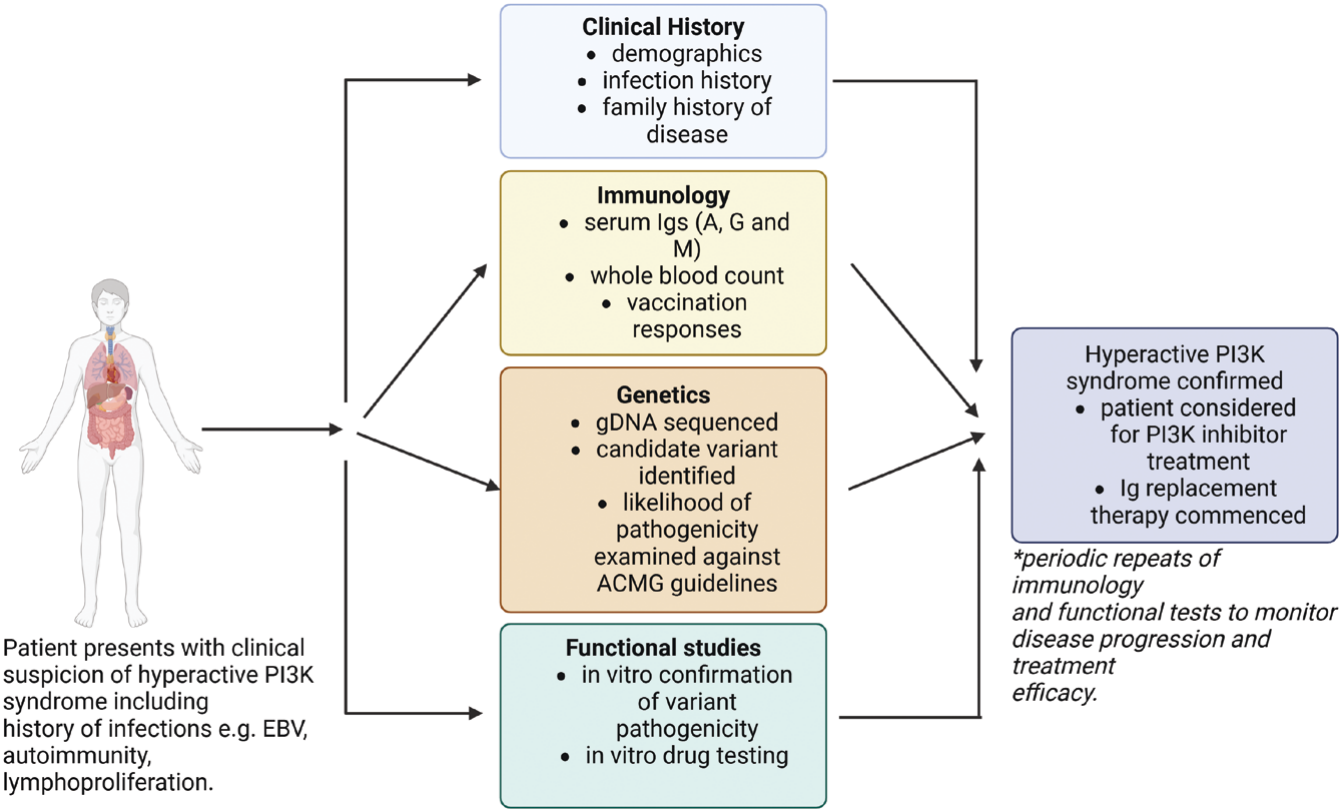
Schematic of the clinical and laboratory investigations used for the diagnosis of APDS and APDS-L. Diagnosis of disease requires thorough evaluation of patients current and past clinical history to identify characteristics of disease consistent with APDS and APDS-L, alongside quantitation of serum Ig levels, vaccination responses, and immunophenotyping of peripheral blood immune cells. This will firstly confirm that the patient has an inborn error of immunity and provides a means to identify characteristic immunological patterns that can aid in differentiating between different APDS and APDS-L disease. Further to this genomics is employed identify the affected gene underlying disease, with supporting assays to functionally validate and confirm these genetics lesions as causative of disease. Thereby, providing informative that could stratify a patient for treatment with PI3K inhibitors, or other treatments such as HSCT. Created with BioRender.com ©.

The establishment of diagnostic and treatment protocols for patients with IEIs is challenging due to their rarity and heterogeneity of clinical presentation. Diagnosis requires a detailed clinical history (**Section** Clinical manifestations of PI3K-related PIDs), as well as specialized immunological, genetic, and functional evaluations (**Fig. 2**) to differentiate APDS from other types of PAD. These investigations are typically undertaken at the time of referral for diagnostic work-up of patients suspected of having PAD. Often these tests are repeated periodically during the course of clinical care for monitoring disease progression and treatment efficacy (**Fig. 2**). In this section, we provide an overview of the tests employed to differentiate between genetic defects that result in hyperactive and underactive PI3K activity, the characteristic laboratory findings for hyperactive PI3K disorders, and discuss the limitations of these tests.

### Serum immunoglobulin levels and vaccination responses

Quantification of serum levels of Ig isotypes, IgG, IgA, and IgM, is fundamental for the diagnosis of an antibody deficiency and provides the basis for treatment with immunoglobulin replacement therapy. Most patients with APDS1 and APDS2 present with normal/increased IgM and reduced IgG and IgA (**Table 3**) [38, 39, 41–44], compared to SYK GOF patients who present with a decreased IgG and M (**Table 3**). In contrast, in patients with either p85α, PIK3CG, p110δ, or PTEN deficiencies all Ig isotypes are decreased [30–32, 42, 45–47, 49, 50, 60], whereas these are normal or decreased in SLP76 deficient patients [51–53]. These observations illustrate that serum Ig levels alone cannot discriminate between hyperactive or underactive PI3K signalling. Due to this ambiguity, further immunological evaluation of peripheral blood immune cell numbers and proportions is required to confirm a patient has an IEI and differentiate between different forms of immunodeficiency e.g. innate, humoral, cellular, or combined immunodeficiency [1, 5, 83, 84].

**Table 3.** Characteristic immunophenotypic features of PI3K hyperactivation syndromes

Immunological features	APDS1	APDS2	APDS-L	SYK GOF
Serum IgG levels				
IgG	↓ [19, 20, 25, 26]	↓ [25, 26, 35-39, 41-44]	↓ [4,7-4,9, 60]	↓ [50]
IgA	↓[19, 20, 25, 26]	↓ [25, 26, 35-39, 41-44]	↓ [4,7-4,9, 60]	NR
IgM	Normal/↑ [19, 20, 25, 26]	Normal/↑ [25, 26, 35-39, 41-44]	↓ [4,7-4,9, 60]	↓ [50]
Bone Marrow				
Pre-B cells	↑ [21, 28, 40, 7,9]	Normal [40]	NR	NR
Pro-B cells	Normal [21, 28, 40,79]	↑ [40]	NR	NR
Mature B cells	↓ [21, 28, 40,79]	Normal [40]	NR	NR
Peripheral blood				
B cells (total)	Normal/↓ [4, 19, 21, 28, 40, 80]	Normal/↓ [35-44]	↑ [48,49, 60]	NR
Transitional	↑ [4, 19, 21, 28, 40, 80]	↑ [35-44]	↑ [4,7-4,9, 60]	NR
Naive	↓ [4, 19, 21, 28, 40, 80]	↓ [35-44]	NR	NR
Unswitched memory	↓ [4, 19, 21, 28, 40, 80]	↓ [35-44]	NR	NR
Class switched (IgG^+^ and IgA^+^) memory	↓ [20, 21, 28, 40]	↓ [35-44]	↓ [4,7-4,9, 60]	NR
CD3^+^ T cells	Normal/↑ [20]	Normal/↑ [37]	Normal [4,7-4,9, 60]	NR
CD4: CD8 ratio	Reversed [23]	Reversed [35-39, 41-44]	Reversed [4,7-4,9, 60]	Reversed [50]
CD4^+^ T cells (total)	↓ [19, 20, 22-24, 40]	↓ [35-44]	↓ [4,7-4,9, 60]	↓ [50]
Naïve (T_N_)	↓ [22, 40]	↓ [35-44]	Normal [4,7-4,9, 60]	↑ [50]
Central Memory (T_CM_)	↑ [22, 40]	Nor mal [35-44]	Normal/↓ [4,7-4,9, 60]	NR
Effector Memory (T_EM_)	↑ [22, 40]	↑ [35-44]	Normal/↓ [4,7-4,9, 60]	NR
T helper 17	NR	NR	NR	↑ [50]
T follicular helper cells (Tfh)	↑ [22, 40, 49]	Normal [35-44]	NR	NR
Regulatory T cells (Tregs)	Normal [22, 40]	Normal [35-44]	Normal [60]	NR
CD8^+^ T cells (total)	↑ [19, 20, 23, 40]	↑ [35-44]	↑ [4,7-4,9, 60]	NR
Naïve (T_N_)	↓ [19, 20, 23, 29, 40]	↓ [35-44]	Normal [4,7-4,9, 60]	NR
Central Memory (T_CM_)	↑ [19, 20, 23, 29, 40]	↑ [35-44]	Normal [4,7-4,9, 60]	NR
Effector Memory (T_EM_)	↑ [19, 20, 23, 29, 40]	↑ [35-44]	Normal [4,7-4,9, 60]	NR
Effector Memory CD45RA revertant (T_EMRA_)	↑ [19, 20, 23, 29, 40]	↑ [35-44]	Normal [4,7-4,9, 60]	NR
Senescent				
CD57^+^	↑ [20, 23, 29, 81]	↑ [35-39, 41-44]	NR	NR
PD-1^+^	↑ [23, 29, 81]	NR	NR	NR
KLRG1^+^	↑ [23, 29, 81]	NR	NR	NR
2B4^+^	↑ [23, 29, 81]	NR	NR	NR
NK cells (total)	Normal [23, 8,2]	Normal/↓ [35-39, 41-44]	Normal [4,7-4,9, 60]	NR

Based on reported proportions of immune cells in peripheral blood. NR, Not reported.

Furthermore, as antibody-deficient patients are known to exhibit impaired responses to vaccination, antibody titres post-challenge with protein and polysaccharide vaccines should be considered with consideration that these investigations should be undertaken prior to the start of Ig replacement therapy, as this can confound results. Furthermore, in some countries including Australia, demonstration of impaired vaccination responses is required for access to immunoglobulin replacement therapy [85].

### Immunophenotypic alterations in peripheral blood

In-depth immunophenotypic evaluation of lymphocyte subset numbers and proportions can establish patterns associated with defined clinical and genetic phenotypes [21, 83, 84]. In addition, these analyses can pinpoint which stage of immune cell development, differentiation, or maturation is affected. This information provides a mechanistic understanding of disease pathophysiology, as well as crucial insights into immunobiology. The patterns here can guide genomics towards a particular gene of interest. Below we outline the immunophenotypic characteristics in hyperactive PI3K disorders.

### B cells

Examination of the B-cell compartment in the bone marrow is confined to APDS. Here, increases in pre-B cells are seen in APDS1 [21, 40], whereas increased pro-B cells are identified in APDS2 [40], with reductions in mature recirculating B cells found in both diseases (**Table 3**) [21, 40]. These findings thus pinpoint the stage at which B-cell development is affected in each disease.

While B-cell lymphopenia is a characteristic feature of APDS 1 and 2 [19, 20, 22–24], it is most frequently reported in APDS2 [26], which differentiates these diseases from PTEN deficiency where no B-cell lymphopenia is observed. Reduced switched (IgA^+^/IgG^+^) memory B cells and increased transitional B cells are a reported characteristic of APDS1, APDS2, and APDS-L (**Table 3**) [19–21, 28, 49, 80]. Additionally, alterations in the B-cell compartment including reduced naïve and unswitched memory cell proportions, are observed in APDS1 and APDS2 but not APDS-L [19–21, 28, 49, 80]. To date, the characteristics of the B-cell compartment have not been reported for SYK GOF.

### CD4^+^ T cells

Extensive examination of the T-cell compartment has also been undertaken in APDS1, APDS2, and APDS-L but not SYK GOF. In these diseases, CD4^+^ T-cell lymphopenia and increased CD8^+^ T-cell proportions are commonly reported accounting for the demonstration of reverted CD4:CD8 ratios (**Table 3**). To date, subsetted CD4^+^ and CD8^+^ T-cell frequencies have not been reported for SYK GOF; thus, from hereon we will concentrate on APDS1, APDS2, and APDS-L.

In both forms of APDS, CD4^+^ T-cell lymphopenia is a result of reduced naïve CD4^+^ T cells, while no change in naïve CD4^+^ T cells is identified in APDS-L, differentiating it from APDS1 and APDS2 (**Table 3**) [22, 23]. Further exploration of the memory compartment shows a characteristic increase in central memory (T_CM_) and effector memory (T_EM_) in APDS1 [22, 40], whereas only T_EM_ frequencies are increased in APDS2 [35–44]. In comparison, the memory compartment as a whole is normal or reduced in APDS-L [47–49, 60]. Another distinguishing feature of APDS1 is the increase in proportion of circulating Tfh [22, 40, 49], which is shown to be normal in APDS2 [35–44]. However, a commonality between all three disorders is the unaltered number of Tregs in peripheral blood (**Table 3**) [22, 35–44, 60].

### CD8^+^ T cells

Increased CD8^+^ T-cell numbers are characteristics of APDS1, APDS2, and APDS-L [19, 20, 23, 35–44, 47–49, 60]. In both APDS1 and APDS2, this is due to increased proportions of all memory populations (T_CM_, T_EM,_ and T_EMRA_ [effector memory CD45RA revertant]), observed alongside reduced naïve T-cell counts (**Table 3**) [19, 20, 23, 35–44]. These findings support accelerated T-cell differentiation and possibly increased exhaustion/senescence. Two papers by Edwards *et al*. and Wentink *et al*. [23, 29] extended upon the initial finding of upregulated expression of senescence marker, CD57 on CD8^+^ memory T cells in APDS1 [20], by showing that CD8^+^ memory T cells also had increased expression of exhaustion marker PD-1, KLRG1, and 2B4. The former is known to be expressed on exhausted cells and the latter is important for EBV-mediated immunity (**Table 3**) [20, 23, 29]. Upregulation of these molecules gives fundamental insight into the mechanisms underlying dysfunction CD8^+^ T-cell memory and is shown to be an underlying cause of CD8^+^ T-cell exhaustion in chronic infection [23]. The aforementioned markers can also be assessed on CD4^+^ T cells to ascertain the probability that these cells are exhausted.

### NK cells

While adaptive immunity is most researched in these diseases, NK cell numbers have been reported in APDS1. Here, NK numbers have been shown to be reduced in some patients [23, 82] and are phenotypically different from normal NK cells. In APDS1, characteristic increases in expression of exhaustion markers like CD57, PD-1, and KLRG1 [23], in conjunction reduced lytic capacity towards EBV-infected targets are reported, providing insights into NK-cell dysfunction and inability to control EBV (**Table 3**) [23, 82].

These characteristic immunophenotypic patterns can be useful in differentiating between disorders and can be associated with a particular genotype, thereby directing genomic analysis. While frequencies of immune cells and their subsets are most commonly reported, in absolute counts are reported in some cases making comparisons between different studies difficult. Therefore, we argue that it is important to undertake a parallel assessment of both absolute and relative numbers to gain a more complete picture of circulating immune cells in the peripheral blood [96].

### Genetic testing

Genomics is the only definitive avenue for precise diagnosis of APDS and other APDS-L disorders (**Fig. 2**). This technology can identify both the gene (e.g. *PIK3CD* versus *PIK3R1*) and the specific variant causing disease (**Table 1**). The choice of methodology employed varies globally. The methodology used depends on availability either locally, or at a diagnostic or research service nationally, or even internationally, as well as access to funding. In some centres, genomics is supported within the healthcare-supported-diagnostic frameworks, whereas other centres rely on collaboration with research centres for access [1].

While methods can range from targeted gene panels to whole-exome sequencing and whole-genome sequencing, a strong suspicion of the gene causative of disease, as well as accurate up to date clinical and immunological data can narrow down the number of genes investigated. Importantly, where gene defects in a single gene can cause two different clinical and immunological phenotypes e.g. genetic defects in *PIK3R1* can cause APDS2 or p85α deficiency (**Table 1**), variants will be cross-referenced with the variants known to cause disease, their location within the gene, and the known mode of inheritance. For example, while APDS2 is an autosomal dominant disease, p85α deficiency is autosomal recessive disease (**Table 1**) [5]. It is also important where possible to genetically screen family members to determine inheritance, which further supports a family history of disease [1].

Other information that is taken in to account when filtering variants from genomics is the sex of the patient, predicted effect on protein function, and prevalence in the general population, alongside clinical phenotype and immunophenotype to ascertain which identified variant(s) are likely causal of disease. From this assessment, variants are classified according to the American College of Medical Genetics and Genomics (ACMG) guidelines to determine predicted variant pathogenicity [1]. It is important to note that a large number of novel variants and variants of uncertain significance (VUSes; category 3) are frequently identified. In these cases, functional assays, are critical for confirming or refuting variants as causal of disease, and as such are indispensable in VUS resolution. These functional assays can also stratify patients for treatment with targeted therapeutics e.g. PI3K inhibitors like rapamycin and leniolisib [1]. When variant pathogenicity is confirmed, it is important that family members are tested. If the causal variant is identified and in the appropriate mode of inheritance, this could flag the patient for monitoring for emergence of symptoms, identify previously overlooked characteristics of disease for more effective treatment, and could be important for family planning [86].

### Functional and biochemical evaluation of immune signalling pathway activity

Demonstration of alterations in the resting and ligand-induced levels, of phosphorylated proteins, including AKT and ribosomal S6, is a powerful tool in the diagnosis of APDS and APDS-L diseases, enabling stratification of patients for treatment, and furthermore monitoring of treatment efficacy.

Increased p-AKT and p-S6 expression are clearly demonstrated at resting and upon stimulation in B and T cells of patients with APDS1 [19, 20, 87], APDS2 [35, 37, 38], and APDSL [48,49,60] (**Table 4**). This biochemical finding establishes hyperactivity of the PI3K-AKT-mTOR-S6 pathway and stratifies these patients as candidates for treatment with PI3K inhibitors. *In vitro*, testing of patient’s cells in the presence of mTOR inhibitor rapamycin or p110δ specific inhibitor, leniolisib, have been shown to normalize PI3K pathway function [20, 87], which has also been confirmed when comparing patients pre- and post-treatment with these agents [87]. Therefore, as well as establishing the functional consequence of variants, *in vitro* testing can predict treatment efficacy in normalizing pathway activity and immune cell phenotype and function, as well as monitor therapeutic effect in treated patients [87].

**Table 4.** Characteristic outcomes of *in vitro* functional tests for hyperactive PI3K disorders

Functional and biochemical tests	APDS1	APDS2	APDS-L	SYK GOF
Proliferation				
B cells	Normal/↑ [20, 21]	Normal/↑ [35, 40]	NR	NR
CD3^+^ T cells (total)	↓ [20]	NR	Normal/↓ [35]	NR
CD4^+^ T cells (total)	NR	↓ [35]	NR	NR
CD8^+^ T cells (total)	Normal [20, 23]	↓ [29, 35]	NR	NR
**Signalling**				
PI3K pathway function				
p-AKT (Thr308)				
CD3^+ ^T cells (blasts)	NR	↑ [35, 37]	↑ [48, 49]	NR
CD4^+ ^T cells	NR	↑ basal and stimulated [35]	NR	NR
B cells	NR	↑ basal and stimulated [38]	NR	NR
p-AKT (Ser473)				
CD3^+ ^T cells (blasts)	↑ basal and stimulated [20, 49, 8,7, 88]	↑ basal and stimulated [35, 37]	↑ [48, 49]	NR
CD4^+ ^T cells	↑ stimulated [19, 20]	NR	NR	NR
CD8^+ ^T cells	↑ stimulated [19, 20]	NR	NR	NR
B cells	↑ basal and stimulated [88]	↑ basal and stimulated [38]	NR	NR
p-S6 (235/236)				
CD3^+ ^T cells (blasts)	↑ basal and stimulated [20, 49, 88]	↑ [35, 37]	↑ [48, 49]	NR
B cells	↑ basal and stimulated [88]	↑ basal and stimulated [38]	NR	↑ [5,6]
p-S6 (240/244)				
CD3^+ ^T cells (blasts)	↑ [8,7]	↑ basal and stimulated [37]	NR	NR
pFoxo1/3a (Thr32/34)				
CD3^+ ^T cells (blasts)	NR	↓ [37]	↓ [48]	NR
Other signalling pathways				
Total PBMC				
p-ERK (Thr202/Tyr204)	NR	NR	↑ [48]	↑ [50]
NFκB	NR	NR	NR	↑ [50]

Note that results for phosphoprotein is based on examination in primary immune cells from patients not overexpression in human cell lines. NR, Not reported.

Importantly, although less widely examined, p-AKT and p-S6 have been shown to be reduced in diseases including p110δ, p110γ, and SLP76 deficiency showing underactive PI3K activity [32–34, 51]. This is important as these patients are unlikely to benefit from PI3K inhibitor treatment, and this biochemical evidence would prevent patient overtreatment with these agents. In fact, a recent study by Edwards *et al*. recently provided an explanation for the previous lack of efficacy of mTOR inhibitor rapamycin in their patient due to abolished PI3K pathway activity [51]. This information could have prevented unnecessary patient treatment with this drug.

Assays examining PI3K function are powerful in the establishment of variant pathogenicity, as well as stratifying patients for treatment with PI3K inhibitors. In addition, as shown by Rao *et al*. these assays can be used to assess the dosing of patients with PI3K inhibitors, like leniolisib [87]. Therefore, these assays will prove useful in establishing when the therapeutic dose has been established, and for monitoring of treatment effectivity long term.

Unfortunately, there is a lack of consensus on which marker to use for assessment (p-AKT versus p-S6) (**Table 4**). However, while p-S6, in particular, has demonstrated robustness in identifying overactivity and underactivity of the PI3K pathway, use of this test is at present largely confined to research laboratories due to the high level of skill and time required to reproduce findings. As such, firstly a decision on the correct marker to examine, as well as more standardized and high-throughput methodology is required to facilitate more widespread use of this assay in diagnostic laboratories and to cement this read-out as the go-to test for hyperactive PI3K disorders.

## Additional tests

EBV and CMV viremia are known to be presenting features of APDS in up to 50% of patients [58]. Therefore, while not documented in the literature monitoring of reactivation of both viruses might be advisable as these viruses ensure that viral control is being maintained, especially as if left unchecked this can cause viral-induced diseases including malignancies like Burkitt’s lymphoma. Furthermore, as the PI3K pathway is known to be exploited by herpesviruses, it may be advisable to monitor viral reactivation in patients receiving PI3K pathway-modulating agents to ensure that immune control is maintained and to enable timely anti-viral treatment before viral-induced disease is established.

## Therapeutic strategies and treatment options for PI3K-related diseases

### Immunoglobulin replacement therapy and immune modulatory treatment

The majority of the APDS patients have an antibody deficiency and/or a poor vaccination response to polysaccharides and are therefore treated with immunoglobulin replacement therapy (IRT) often in combination with antibiotic prophylaxis [36, 59, 89]. This treatment has been reported to reduce the RTI, but it does not prevent infections with herpes viruses, nor the development of autoimmune and autoinflammatory complications, nor lymphoproliferation [24, 27]. Importantly, despite optimal RTI treatment, progression of bronchiectasis occurred in some APDS patients [24, 36, 89]. The autoimmune and inflammatory complications are most often treated with immunosuppressive treatment. A study from the ESID registry showed that most of the patients showed clinical benefits from corticosteroids, or to other immunosuppressive drugs like azathioprine, mycophenolate, or cyclosporine [25]. Rituximab treatment also results in clinical benefit [25]; however, this can lead to a sustained B-cell lymphopenia [24]. Patients with PHTS hardly have recurrent infections, and half of the PHTS patients with an immunodeficiency (4/8) required treatment with IRT and or antibiotics. For the described patients with the SYK GOF variants, inflammation is the most prominent clinical features, and therefore, all patients received one or more immunomodulatory treatment including anakinra, 5-aminosalicyclin acid-based therapy, azathioprine, CHOP, CSTD, cyclosporine A, rituximab, tacrolimus, and vedolizumab [50]. In addition, five patients were treated with IRT and antibiotics.

### Hematopoietic stem cell transplantation

HSCT is still the only curative treatment for the immune system in APDS. Recently, an international retrospective cohort study on 57 APDS1 and APDS2 patients who received HSCT was published [83]. All of these patients had an indication for HCST, which included haematologic malignancy (26%), non-malignant proliferation (49%), autoimmune cytopenia’s (14%), chronic or recurrent infections (46%), end-organ damage (28%), or a combination indication (53%). The overall survival after 2 years was 86% and did not differ between APDS1 and APDS2, neither in the condition intensity. However, there was a high risk of graft failure or need for unplanned donor cell infusions, which is probably related to the intensity of the host lymphodepletion, rather than myeloablation. Importantly, especially the use of mTOR inhibitors in the first year post-HSCT increased the risk of graft failure from 17% to 42%. This is likely caused by the increased survival and reduced T-senescence of the host lymphocytes upon the use of mTOR inhibitors and might also be the case for the use of other PI3K-pathway inhibitors. So far, no report has been published on patients with APDS-L or SYK GOF variants that received a HSCT, although mice studies indicated that HSCT could be a potential treatment option [50].

### PI3K pathway modulators

Over the past 10 years, seven clinical trials (**Table 5**) have been undertaken in paediatric patients ranging from 1 to 18 years, as well as adults up to 75 years to assess the toxicity and therapeutic efficacy of mTOR (rapamycin) and PI3K (leniolisib) inhibitors in patients with APDS1 and APDS2 (**Table 5**).

**Table 5:** Clinical trials investigating mTOR and PI3K inhibitors as treatment for APDS

Drug	Trial identifier	Phase	Target population	Name of trial/study	Status	References
Nemiralisib	NCT02593539	2	Adults (18 Years and Older)	An Open-label, Single Arm Study to Investigate the Safety, Pharmacokinetics and Pharmacodynamics of Repeat Doses of Inhaled Nemiralisib in Patients With APDS/PASLI	Completed 2020-06-04	
Rapamycin	NCT03383380	1/2	Children/ Adults (up to 18 years)	Rapamycin Treatment for Activated Phosphoinositide 3-Kinase δ Syndrome	Completed 2023-11-30	[20, 49] [19]
Leniolisib (CDZ173)	NCT02859727	2/3	Children/ Adults/ Older Adults (12 to 75 years)	Extension to the Study of Efficacy of CDZ173 in Patients With APDS/PASLI	Active, not recruiting	
Leniolisib (CDZ173)	NCT06249997	3	Children/ Adults/ Older Adults (12 to 75 years)	An Open-Label Study to Assess the Safety & Efficacy of Leniolisib in Japanese Patients With APDS	Recruiting	
Leniolisib (CDZ173)	NCT05438407	3	Children (4 to 11 years old)	Pediatric Patients Aged 4 to 11 Years With APDS	Active, not recruiting	
Leniolisib (CDZ173)	NCT05693129	3	Children (1 to 6 years old)	Pediatric Patients Aged 1 to 6 Years With APDS	Recruiting	
Leniolisib (CDZ173)	**NCT02435173**	2/3	Children/ Adults/ Older Adults (12 to 75 years)	Study of Efficacy of CDZ173 in Patients With APDS/PASLI	Completed 2021-08-16	[87, 91] [92]

### mTOR inhibition

Already in 2014, Lucas *et al*. showed that GOF mutations in *PIK3CD* result in increased phosphorylation of AKT and hyperactivation of the downstream kinase mTOR. They showed that the T-cell defect *in vitro* could largely be rescued by the mTOR inhibitor rapamycin. Importantly, they administered rapamycin to an APDS patient with extreme lymphoproliferation and showed a reduction of CD8^+^ T cells and a reduction in hepatosplenomegaly and lymphadenopathy [20]. More recent case studies have shown in larger numbers of patients that rapamycin also known as sirolimus has a positive effect in the majority of the patients (*n* = 48), especially in reducing the lymphadenopathy and splenomegaly [25]. However, the effect on infections, autoimmune cytopenia’s, and enteropathy is limited. The treatment with sirolimus is complicated by complex pharmacokinetics which can be influenced by many different factors and should be closely monitored [93]. Furthermore, in patients with solid organ transplantation, the treatment with sirolimus is associated with adverse side effects including hyperglycaemia, dyslipidaemia, cytopenia’s, nephrotoxicity, stomatitis and skin eruptions, poor wound healing, and pneumonitis [94]. For APDS patients the long-term side effects still need to be studied, but a study from the ESID registry showed that of 26 patients treated with sirolimus, two patients need required complete cessation, and three patients paused the treatment because of adverse side effects [26]. Moreover, new mTOR inhibitors like everolimus, temsirolimus, sapanisertib, and RapaLink-1 are currently tested for use in cancer treatment, but the use of these newer mTOR inhibitors has not yet been tested in APDS patients [93]. The use of mTOR inhibitors in APDS-L and SYK GOF patients is limited, only one case report in a patient with APDS-L treated with sirolimus and ustekimab showed improvement of immunological parameters, gastrointestinal complains, and improvement of general well-being [95].

### Specific PI3K inhibitors

While mTOR inhibitors target the PI3K signalling downstream of the PI3 kinase, more specific PI3K inhibitors were also developed for use in oncology. In 2017, the first clinical trial for the use of the PI3K inhibitor leniolisib in six patients with APDS was published in which the safety, pharmacokinetics, and effect on lymphoproliferation and immune dysregulation were studied [87]. After 12 weeks, all patients showed reduced lymphoproliferation and patients reported improved well-being. Recently, a 6-year follow-up from the same patients was published [91]. Long-term improvement of the lymphocyte subsets was observed, as well as decreased lymph node, liver, and spleen size. Of the five patients receiving IRT at base line, two patients discontinued IRT and two patients reduced the dose. All patients reported improved health-related quality of life. Other PI3K inhibitors like nemiralisib (**Table 5**), seletalisib, and idelalisib have not been proven to be effective in APDS or show considerable side effects [93].

## Challenges and future perspectives

APDS is a good example of how advances in genetic testing and the availability of small molecule inhibitors have led to the description of more than 200 patients, and an FDA-approved targeted treatment only 10 years after the first description of the genetic defect. So far, genetic variants in APDS1, APDS2, and APDS-L are known to result in PI3K hyperactivation, but in the future, other genetic defects like SYK GOF can likely be added to the PI3K hyperactivation syndromes. Especially, since genetics testing is still becoming cheaper and easier accessible, especially in lower-income countries. The challenge remains, however, to functionally validate the VUSes.

The clinical phenotypes of APDS1, APDS2, APDS-L, and SYK GOF have overlapping clinical and immunological features but are also very heterogeneous. They do not only result in an immunodeficiency but also immune dysregulation leading to severe complications like lymphoproliferation, autoimmunity, enteropathy, and malignancies. This stresses the importance of multi-disciplinary patient care but also highlights the major clinical challenge to prevent these severe complications. The large retrospective study of Dimitrova et al. showed that, the only curative treatment option, HSCT, is only performed in patients with a clinical indication for HSCT (e.g. malignancy) and not based on the genetic diagnosis alone [90]. The specific PI3K delta inhibitor, leniolisib, seems to be a promising therapy for APDS to normalize the immunological phenotype and to reduce lymphoproliferation. However, this treatment is not yet available for all APDS patients. Moreover, it is not registered for the use in children so far, while treating APDS early in life might be the only way to prevent the severe complications. In addition, there are also concerns about the costs, which might limit the number of APDS patients that have access to the treatment. So, a major future challenge of APDS remains to find an affordable treatment that can prevent severe complications.

## Data Availability

There are no novel data generated for this review article.

## Abbreviations

AIHA: Autoimmune haemolytic anaemia
APDS: Activated p110δ syndrome
APDS-L: APDS-like
BCR: B-cell receptor
CMV: Cytomegalovirus
EBV: Epstein Barr Virus
GM-CSFR: Granulocyte-macrophage colony-stimulating factor receptor
GOF: Gain of function
GPCR: G-coupled receptors
HSV: Herpes simplex virus
IEI: Inborn Errors of Immunity
IGR-1R: insulin-like growth factor 1 receptor
IRT: immunoglobulin replacement therapy
ITP: immune thrombocytopenia purpura
PAD: predominantly antibody deficiency
PHTS: PTEN hamartoma tumour syndrome
PI3K: phosphoinositide-3-kinase
PTEN: phosphatase and tensin homolog
RTK: receptor tyrosine kinase
SHIP: SH2-containing inositol 5’phsphatase
SHORT: Short stature Hyperextensibility of joints and/or hernias, Ocular depression, Rieger anomaly and delays of Tooth eruptions
SYK: Spleen tyrosine kinase
TCR: T-cell receptor
TLR: Toll-like receptor
VZV: Varicella zoster virus
